# Calcium-sensing receptor AcCaS regulates chloroplast immunity in kiwifruit by competitively binding with Ca^2+^ or the *Psa* effector

**DOI:** 10.1093/hr/uhaf230

**Published:** 2025-09-03

**Authors:** Rui Li, Yali Zhang, Xiaofei Du, Xinxin Wang, Wei Liu, Lili Huang

**Affiliations:** State Key Laboratory of Crop Stress Resistance and High-Efficiency Production, College of Plant Protection, Northwest A&F University, Yangling, Shaanxi 712100, China; State Key Laboratory of Crop Stress Resistance and High-Efficiency Production, College of Plant Protection, Northwest A&F University, Yangling, Shaanxi 712100, China; State Key Laboratory of Crop Stress Resistance and High-Efficiency Production, College of Plant Protection, Northwest A&F University, Yangling, Shaanxi 712100, China; State Key Laboratory of Crop Stress Resistance and High-Efficiency Production, College of Plant Protection, Northwest A&F University, Yangling, Shaanxi 712100, China

## Abstract

Activating chloroplast immunity to enhance host resistance offers a novel and sustainable approach for the effective control of kiwifruit bacterial canker. Chloroplasts serve as a central hub for ROS, SA, and Ca^2+^ signaling. As a chloroplast-localized protein, CaS participates in Ca^2+^-signaling pathways. However, the mechanisms underlying CaS-mediated immune regulation and whether to be attacked by pathogens remain unclear. Here, we created AcCaS-overexpressing transgenic plants, then we found that AcCaS activates chloroplast reactive oxygen species (ROS) bursts and enhances resistance against *Pseudomonas syringae* pv. *actinidiae* (*Psa*). Mutational analysis revealed that the chloroplast transit peptide (cTP) of AcCaS is essential for its immune function, and deletion of cTP abolished ROS production and disease resistance. Yeast two-hybrid reveals that *Psa* employs the effector HopAU1 targets AcCaS in kiwifruit. Luciferase complementation imaging, and microscale thermophoresis assays identified Asn-121 of AcCaS as the critical residue mediating both HopAU1 binding and Ca^2+^ sensing. Strikingly, molecular modeling and competitive binding experiments showed that HopAU1 directly occupies the Ca^2+^-binding site at Asn-121, thereby blocking calcium signaling and suppressing chloroplast immunity. In summary, this study uncovers that *AcCaS* enhances resistance against *Psa* by activating chloroplast ROS and binding with Ca^2+^. The Asn-121 residue plays a pivotal role in Ca^2+^-binding and HopAU1-mediated immune suppression, as mutations at this site abolish both activities. These findings revealed the battle of chloroplast Ca^2^ signaling in plant–pathogen conflicts and provide a mechanistic basis for engineering AcCaS-centered resistance in kiwifruit.

## Introduction

Kiwifruit (*Actinidia* spp.) is a widely cultivated horticultural crop with high economic value, rich in vitamin C, carotene, amino acids, and other essential nutrients [1, 2]. However, kiwifruit bacterial canker (KBC), caused by *Pseudomonas syringae* pv. *actinidiae* (*Psa*), is the most devastating disease affecting kiwifruit production. Symptoms include leaf spots, flower rot, and trunk exudation. In severe cases, it can lead to branch dieback or even tree death [3]. KBC poses a significant threat to the kiwifruit industry due to its broad adaptability, widespread impact, and frequent outbreaks. Its persistent effects and the difficulties in prevention and control further exacerbate the problem [3–7]. Tackling this issue is crucial for promoting the industry’s long-term health and sustainable growth. Conducting in-depth research on the interactions between *Psa* and kiwifruit is crucial for developing innovative, eco-friendly, and sustainable strategies for disease prevention and management.

Chloroplasts serve as key organelles mediating photosynthesis and function as critical signaling hubs in plant immune responses [8]. Chloroplasts produce critical defense-related molecules such as cytoplasmic Ca^2+^ waves, reactive oxygen species (ROS), phytohormone precursors such as salicylic acid (SA) and jasmonic acid (JA) [9]. In the co-evolutionary conflict between plants and pathogens, chloroplasts have become a prime target for pathogens. For example, HopI1 from *P. syringae* directly acts on chloroplasts, remodeling thylakoid membranes and suppressing SA accumulation and SA-dependent defense responses [10]. HopN1 is a cysteine protease with proteolytic activity that targets chloroplasts by degrading PsbQ in photosystem II, thereby reducing photosynthetic electron transport and ROS production [11, 12]. Additionally, the effectors such as AvrRps4 and HopK1 require chloroplast localization to suppress pattern-triggered immunity (PTI) responses [13]. The RXLR effector RXLR31154 from *Plasmopara viticola* promotes pathogen colonization by stabilizing PsbP in chloroplasts, activating the singlet oxygen signaling pathway, and reducing hydrogen peroxide (H_2_O_2_) accumulation [14]. Although chloroplast-mediated immune mechanisms have been extensively studied in various plants, the molecular basis of chloroplast immunity in kiwifruit remains poorly understood.

Calcium-sensing receptor (CaS) is a key regulatory protein in photosynthesis, stomatal closure, and responses to abiotic stresses such as drought, high temperatures, and salinity [15, 16]. Transgenic expression of OsCAS in *Arabidopsis* enhanced drought tolerance, demonstrating the functional conservation and significance of CaS in plant drought resistance mechanisms [17]. It has also been implicated in a chloroplast-mediated retrograde signaling pathway in green algae, suggesting a calcium ions (Ca^2+^) and CO_2_-signaling mechanism dependent on photosynthetic biota [18, 19]. CaS can precisely sense changes in intracellular and extracellular Ca^2+^ levels. When Ca^2+^ concentrations increase, CaS is activated, thereby initiating downstream signal transduction cascades [20, 21]. In *Arabidopsis*, *CaS* is a single-copy gene. CaS produces a protein made up of 387 amino acids [15]. T-DNA insertion mutants of *Arabidopsis* confirmed that CaS is essential for inducing stomatal closure and controls plant innate immunity by regulating SA biosynthesis [22]. In *Arabidopsis cas-1* mutant, studies revealed increased the virulence of *P. syringae* pv. *tomato* (*Pst*), suppression of flg22-mediated PTI, and delayed or suppressed AvrRpt2-mediated effector-triggered immunity (ETI) [22]. Additionally, SsITL from *Sclerotinia sclerotiorum* suppressing CaS-mediated SA accumulation and promoting fungal infection [23]. Overall, the roles and underlying mechanisms of CaS in plants remain limited. Particularly, the mechanism by which CaS, responds to Ca^2+^ to trigger plant immunity remains unclear.

In this study, we characterized *AcCaS* that enhances plant immunity by promoting the chloroplast ROS burst and binding with Ca^2+^. The chloroplast localization of AcCaS is critical for its activation of immune responses. A *Psa* effector HopAU1 enhances the infection by competitively binding to the Asn-121 residue of AcCaS with Ca^2+^ on chloroplasts. We identified Asn-121 is critical for Ca^2+^-binding and HopAU1-mediated immune suppression. This study uncovers a strategy used by *Psa* to manipulate host immunity and provides insights into the role of AcCaS in mediating kiwifruit disease resistance. The identification of critical sites in AcCaS that contribute to disease resistance provides theoretical support for the rational design of disease-resistant related molecules targeting chloroplast signaling hubs.

## Results

### AcCaS is significantly involved in the kiwifruit–*Psa* interaction

**Figure 1:**
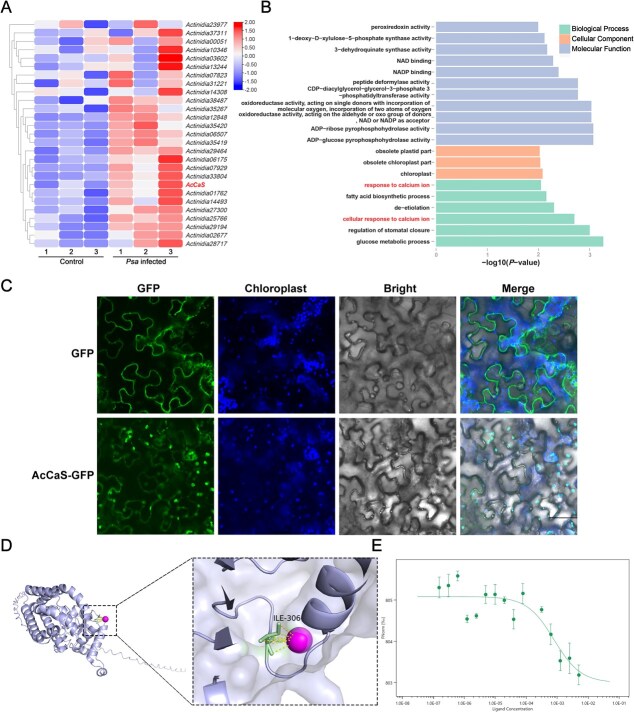
Identification of the AcCaS during *Psa* infection in kiwifruit. (A) Expression profiles of chloroplast related genes in transcriptomic data. The specified scale represents the log_2_ transformation of the standardized gene expression level. (B) Analysis of chloroplast-related genes using GO pathway enrichment. (C) Subcellular localization of AcCaS. *Agrobacterium tumefaciens* carrying GFP and AcCaS -GFP was injected into *N. benthamiana* leaves. The leaf epidermis was observed under confocal microscope FV3000 (ocular: 10×; objective: 20×) at 48 hpi. Bar = 70 μm. Three separate biological studies converge on consistent results. (D) 3D structure of AcCaS and Ca^2+^ by AlphaFold3 modeling. (E) MST assays show that AcCaS^△cTP^ interacts with Ca^2+^. The Ca^2+^ and AcCaS^△cTP^-GST were contained in NT standard capillaries. Three separate biological studies converge on consistent results.

Given the significance of chloroplasts in plant immune processes, we identified a disease-resistant line RH12 and a susceptible line SH14 through disease resistance screening of a diploid hybrid population in kiwifruit (*Actinidia chinensis* var. *chinensis*) [24]. To explore the molecular signaling and regulatory pathways involved in the interaction between kiwifruit and *Psa*, we conducted transcriptome analysis on RH12 and SH14 leaf samples affected by *Psa* at 24 h postinfection (hpi). We focused on investigating the role of chloroplast-associated genes in pathogen resistance. Through analysis of chloroplast-related differentially expressed genes (DEGs) in *Psa*-infected kiwifruits, we identified 27 genes significantly upregulated in RH12 (Table S1, Fig. 1a). Among these, we found that AcCaS exhibits the response to calcium ions (Ca^2+^) (Fig. 1a and b). qRT-PCR analysis further confirmed that *AcCaS* was significantly upregulated upon *Psa* infection. And the expression of *AcCaS* was higher in disease-resistant varieties *A. chinensis* var. *deliciosa* ‘Cuixiang’ (Fig. S1a). This is consistent with the involvement of AcCaS in regulating disease resistance.

CaS is widely present across different species, relatively conserved in evolution, and shares close phylogenetic relationships with species such as *Schima superba*, tea (*Camellia sinensis*), and pear (*Pyrus communis*), providing a reference for the subsequent functional analysis of AcCaS (Fig. S1b). Analogous to *Arabidopsis*, *CaS* is a single-copy gene in kiwifruit genome. AcCaS is composed of 395 amino acids, and domain analysis predicts that its N-terminal region (amino acids 1–70) is a chloroplast transit peptide (cTP), amino acids 198–220 forming a transmembrane region and amino acids 232–346 containing the conserved rhodanese homology domain (RHOD) structural domain (Fig. S1c). To elucidate its function, AcCaS was combined with a green fluorescent protein (GFP) for transient expression. Chloroplast autofluorescence served as a means to identify and localize chloroplasts. In AcCaS-GFP expressing cells, a GFP signal was specifically localized to chloroplasts (Fig. 1c). To characterize the Ca^2+^-binding capability of AcCaS, we employed AlphaFold3 modeling, which predicted that the Ile-306 residue within AcCaS interacts with Ca^2+^ (Fig. 1d). Furthermore, using microscale thermophoresis (MST) analysis, we confirmed that AcCaS^△cTP^ exhibits the capability to bind Ca^2+^ (Fig. 1e).

### AcCaS positively regulates kiwifruit resistance to *Psa*

**Figure 2:**
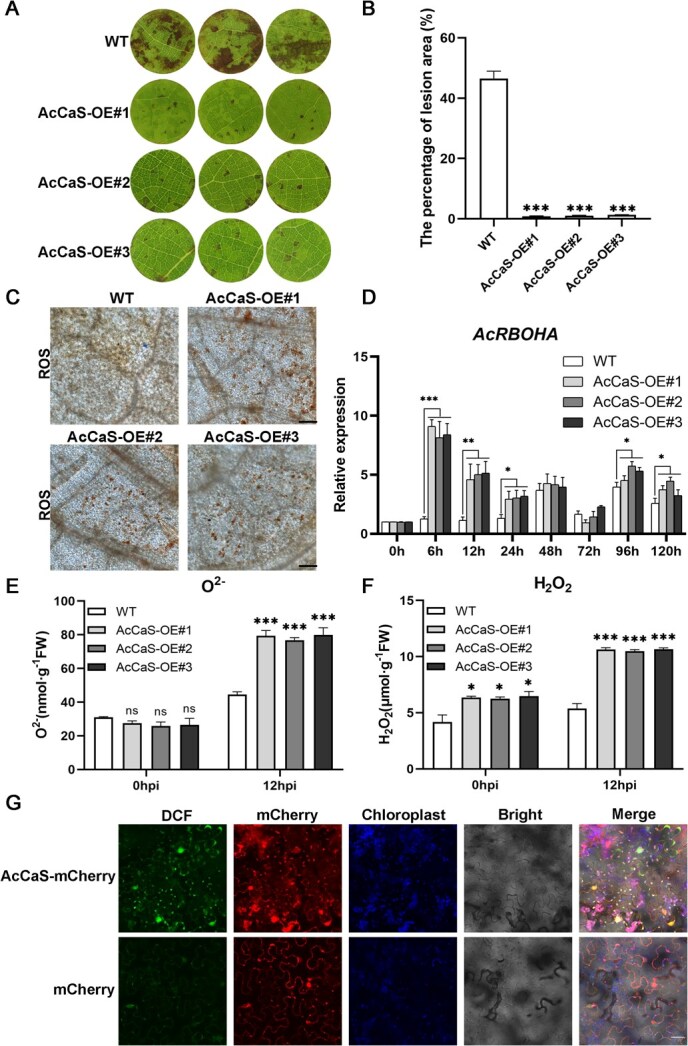
AcCaS confers resistance to *Psa* in kiwifruit. (A) Phenotypic observation of AcCaS-OE or WT kiwifruit leaves at 5 dpi. (B) The lesion area percentage of AcCaS-OE or WT kiwifruit leaves at 5 dpi. (C) The accumulation of ROS in AcCaS-OE or WT kiwifruit leaves at 12 h postinoculation (hpi). (D) Relative expression levels of ROS related gene *AcRBOHA* of AcCaS-OE lines at different times. The relative expression levels were determined by employing the 2^−ΔΔCT^ method. The qRT-PCR values were standardized using *Actin* as the reference gene. (E, F) The content of O^2−^ and H_2_O_2_ in AcCaS-OE lines or WT leaves at 12 hpi. (G) *N. benthamiana* leaves subjected to H2DCF-DA were examined under fluorescence microscopy for DCF signal detection at 32 hpi, after overexpressing AcCaS-mCherry or mCherry. Bar = 50 μm. The error bar represents the variations among five independent replicates. All the difference is statistically significant by Student’s *t*-test: ^*^, *P <* 0.05; ^**^, *P <* 0.01; ^***^, *P <* 0.001; ns, not significant.

To investigate whether AcCaS improves the resistance of kiwifruit against *Psa*, we created transgenic kiwifruit plants overexpressing *AcCaS* to verify the findings. AcCaS was highly overexpressed in transgenic plants, according to western blot (Fig. S2). After inoculation with *Psa*, the infected leaves of AcCaS-OE lines exhibited milder disease progression with a significantly lower lesion area percentage compared to WT at 5 dpi (Fig. 2a and b). This indicates the potential positive regulatory role of AcCaS in kiwifruit–*Psa* interaction process.

In reaction to both biotic and abiotic stressors, plant cells can counteract by bursting with ROS. Following inoculation with *Psa*, we visualized the accumulation pattern of ROS using 3,3′-diaminobenzidine (DAB) and quantified ROS levels. After inoculated with *Psa* for 12 h, AcCaS-OE lines exhibited higher ROS contents compared to WT (Fig. 2c, and  e-f). Furthermore, the expression of ROS-associated genes *AcRBOHA* (*Actinidia10614*) exhibited substantial upregulation at different time points of *Psa* inoculation in AcCaS-OE lines (Fig. 2d). Given that ROS are inherent byproducts of the photosynthetic process and predominantly originate within chloroplasts, we quantified the accumulation of ROS derived from chloroplasts. The compound 2′7′-dichlorodihydrofluorescein diacetate (H2DCF-DA) served as the measuring tool in *Nicotiana benthamiana* leaves overexpressing AcCaS-mCherry. Significantly, AcCaS-mCherry induced a substantial accumulation of ROS within chloroplasts, whereas mCherry did not produce a similar outcome (Fig. 2g). The results showed that AcCaS enhances chloroplast ROS burst to promote immunity.

Additionally, we investigated the expression of defense-related genes *AcNPR1* (*Actinidia29909*), *AcPR1* (*Actinidia38643*), *AcMYC2a* (*Actinidia14806*), and *AcERF1* (*Actinidia09666*) linked to salicylic acid (SA), jasmonic acid (JA), and ethylene (ET) signaling in AcCaS-OE lines and inoculated with *Psa* [22]. qRT-PCR results revealed a significant upregulation of *AcNPR1*, *AcPR1,* and *AcERF1* expression (Fig. S3). These findings suggest that AcCaS may influence SA and ET signaling pathways, potentially enhancing kiwifruit resistance to *Psa*.

### The chloroplast localization of AcCaS is critical for its activation of immune responses

**Figure 3:**
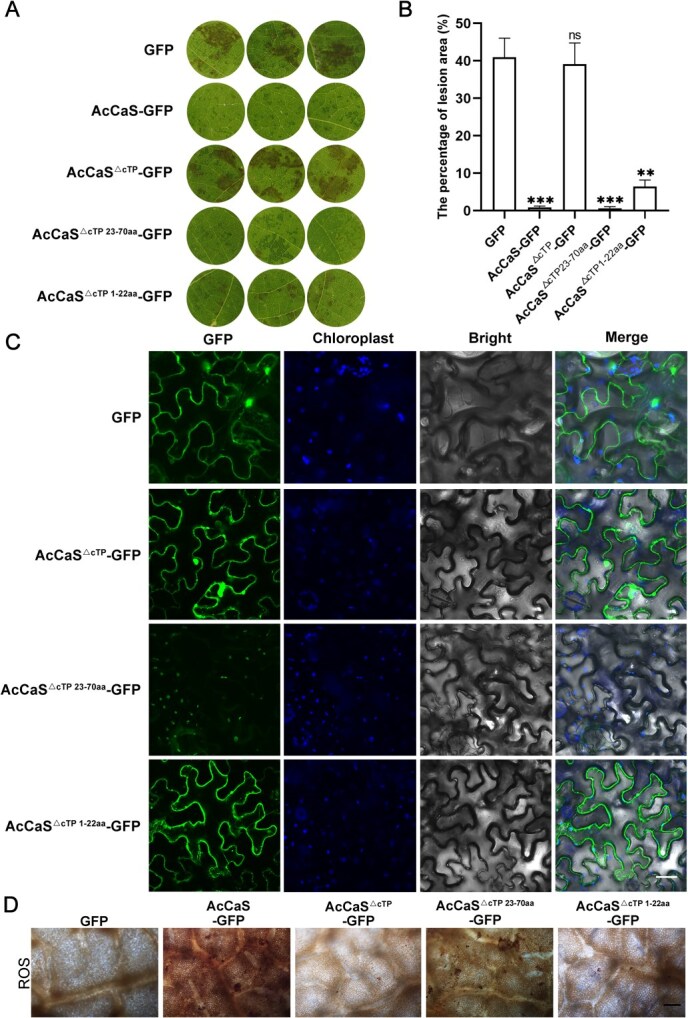
The localization of AcCaS in the chloroplast is crucial for triggering plant immune responses. (A) Phenotypic observation of kiwifruit leaves transiently overexpressing GFP, AcCaS-GFP, AcCaS^△cTP^-GFP, AcCaS^△cTP 1-22aa^-GFP or AcCaS^△cTP 23-70aa^-GFP at 5 dpi. (B) The lesion area percentage of kiwifruit leaves of transiently overexpressing GFP, AcCaS-GFP, AcCaS^△cTP^-GFP, AcCaS^△cTP 1-22aa^-GFP or AcCaS^△cTP 23-70aa^-GFP at 5 dpi. (C) Subcellular localization of AcCaS^△cTP^, AcCaS^△cTP 1-22aa^ and AcCaS^△cTP 23-70aa^. *Agrobacterium tumefaciens* carrying GFP, AcCaS^△cTP^-GFP, AcCaS^△cTP 1-22aa^-GFP or AcCaS^△cTP 23-70aa^-GFP was injected into *N. benthamiana* and the leaf epidermis was observed under confocal microscope FV3000 (ocular: 10×; objective: 20×) at 48 hpi. Bar = 50 μm. (D) The accumulation of ROS in kiwifruit leaves transiently overexpressing GFP, AcCaS-GFP, AcCaS^△cTP^-GFP, AcCaS^△cTP 1-22aa^-GFP or AcCaS^△cTP 23-70aa^-GFP at 12 hpi. The error bar represents the variations among five independent replicates. All the difference is statistically significant by Student’s *t*-test: ^**^, *P <* 0.01; ^***^, *P <* 0.001; ns, not significant.

AcCaS is localized to the chloroplast (Fig. 1c). To investigate the relationship between the chloroplast localization of AcCaS and its role in disease resistance, we used the pCAMBIA1302 overexpression vector to express three cTP-deleted AcCaS variants: AcCaS^△cTP^ (complete deletion), AcCaS^△cTP 1-22aa^ (deletion of residues 1–22), and AcCaS^△cTP 23-70aa^ (deletion of residues 23–70). GFP represents the kiwifruit with the empty vector. The overexpression system performed effectively, as evidenced by the significant increase expression of *AcCaS* at 2 dpi (Fig. S4). After inoculation with *Psa*, the infected leaves of AcCaS-GFP and AcCaS^△cTP 23-70aa^-GFP exhibited milder disease progression and the lesion area percentage was significantly lower than AcCaS^△cTP^-GFP and AcCaS^△cTP 1-22aa^-GFP kiwifruit leaves at 5 dpi (Fig. 3a and b). This indicates the resistance of AcCaS is related to its chloroplast localization.

**Figure 4:**
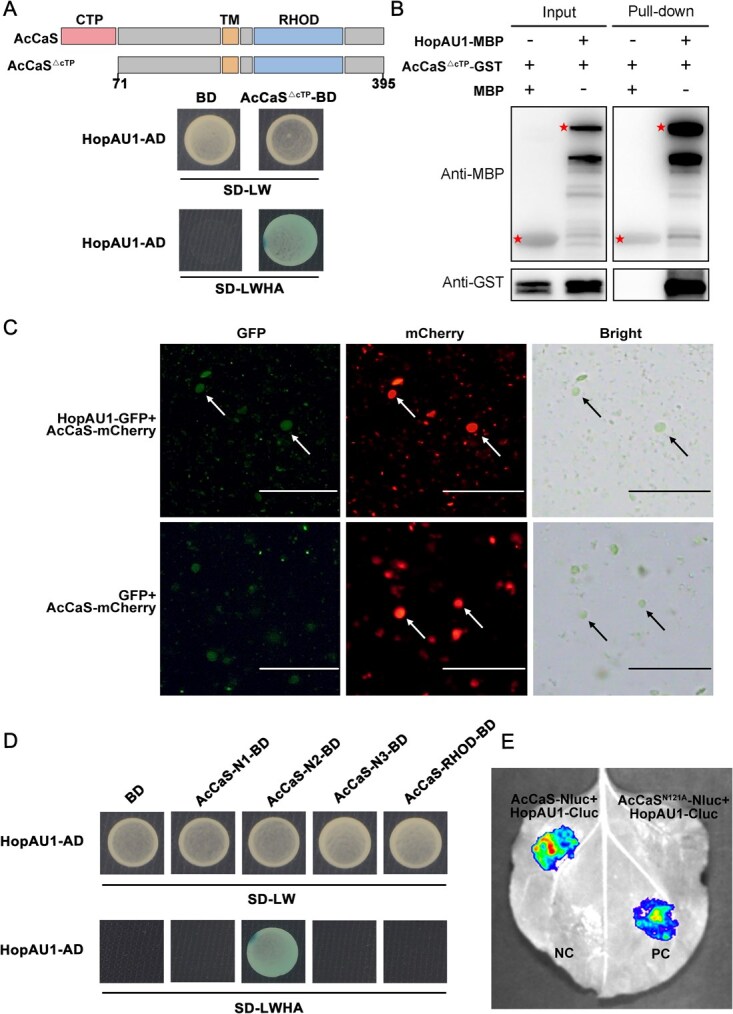
HopAU1 directly interacts with AcCaS on the chloroplast. (A) Yeast two-hybrid (Y2H) analysis of the interactions between HopAU1 and AcCaS. Yeast transformants possessing distinctive constructs were cultivated on SD media devoid of LW (-Leu/-Trp), while those exhibiting LacZ activities were cultured on SD-LWHA (-Leu/-Trp/-His/-Ade) supplemented with X-α-gal. The working concentration of X-α-Gal was 40 μg/ml. (B) Pull-down of AcCaS^△cTP^-GST by HopAU1-MBP. GST was utilized as a control in the negative sense. Asterisks signify the regions of proteins. (C) Chloroplasts derived from *N. benthamiana* transient expression of HopAU1-GFP and AcCaS-mCherry fusion was investigated using epifluorescence and bright field (BR) microscopy. *N. benthamiana* leaves transiently exhibiting GFP and AcCaS-mCherry were analyzed to serve as a control. Arrows indicate the presence of chloroplasts. Bar = 50 μm. (D) Y2H analysis of the interactions between HopAU1 and AcCaS truncation mutants. (E) HopAU1 is unable to interact with the AcCaS^N121A^ in LCI assays. Co-expression of HopAU1-cLuc and AcCaS^N121A^-nLuc in *N. benthamiana* leaves, the imaging was observed with multispectral dynamic fluorescence microscopy (PlantView100) at 2 dpi. *N. benthamiana* leaves transiently expressing HopAU1-cLuc and AcCaS-nLuc were examined as the control. A combination of MdVQ29-nLuc and MdWRKY23-cLuc served as a positive control [25], GFP-nLuc and HopAU1-cLuc were utilized as negative controls.

For determining the subcellular location of AcCaS^△cTP^, AcCaS^△cTP 1-22aa^, AcCaS^△cTP 23-70aa^, we transiently expressed AcCaS^△cTP^-GFP, AcCaS^△cTP 1-22aa^-GFP and AcCaS^△cTP 23-70aa^-GFP in *N. benthamiana*. The green fluorescence signal generated by AcCaS^△cTP 23-70aa^-GFP overlapped with the autofluorescence of chloroplast (Fig. 3c), indicating that the AcCaS^△cTP 23-70aa^ is targeted to the chloroplast. Further, following inoculation with *Psa*, we visualized the accumulation pattern of ROS using DAB. The kiwifruits overexpressing AcCaS-GFP and AcCaS^△cTP 23-70aa^-GFP showed higher ROS levels compared to the GFP, AcCaS^△cTP^-GFP and AcCaS^△cTP 1-22aa^-GFP at 12 hpi (Fig. 3d). This indicates that the disease resistance function of AcCaS requires its chloroplast localization, and the N-terminal 1–22 amino acids are essential for its chloroplast targeting.

### AcCaS directly interacts with HopAU1 on the chloroplasts

We conducted interaction screening between AcCaS and 31 effectors from *Psa*, using the yeast two-hybrid (Y2H) system which revealed a specific physical interaction between AcCaS and HopAU1 (Fig. 4a). HopAU1 can interact with AcCaS *in vivo* [26]. To further explore the interaction mechanism between HopAU1 and AcCaS, we used pull-down assays to investigate their potential direct interaction. In pull-down assay, vectors expressing AcCaS^△cTP^-GST and HopAU1-MBP, as well as control vectors expressing AcCaS^△cTP^-GST and MBP, were coexpressed in *Escherichia coli*, respectively. We found that AcCaS^△cTP^-GST can be purified together with HopAU1-MBP, but not MBP (Fig. 4b).

**Figure 5:**
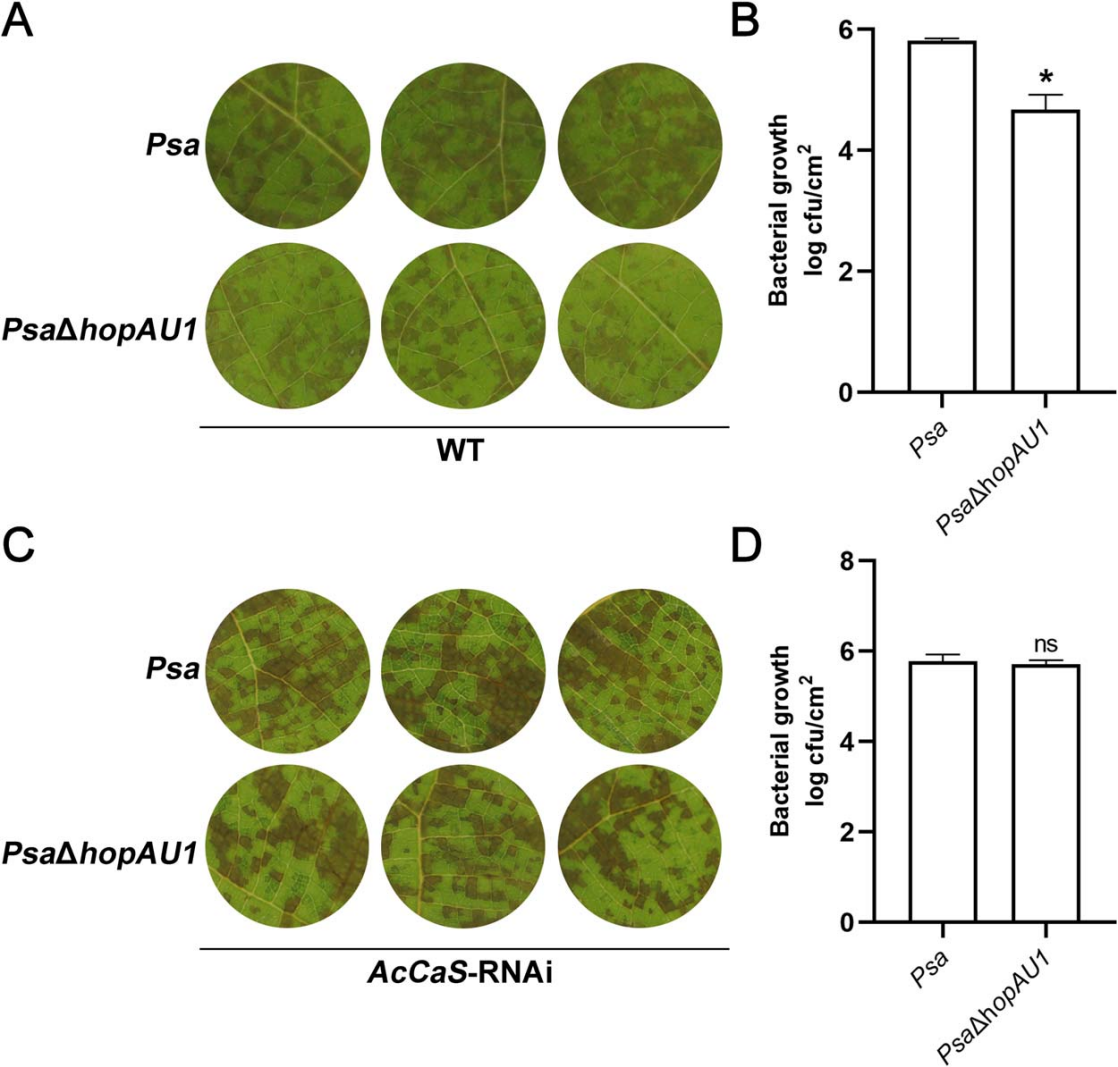
HopAU1 contributes to the virulence of *Psa* relies on AcCaS. (A) Phenotypic observation of kiwifruit leaves inoculated with *Psa* or *Psa*Δ*hopAU1* at 5 dpi. (B) The bacterial growth assays of *Psa* or *Psa*Δ*hopAU1* were performed in kiwifruit leaves at 5 dpi. (C) Phenotypic observation of *AcCaS*-RNAi kiwifruit leaves inoculated with *Psa* or *Psa*Δ*hopAU1* at 5 dpi. (D) The bacterial growth assays of *Psa* or *Psa*Δ*hopAU1* were performed in *AcCaS*-RNAi kiwifruit leaves at 5 dpi. The error bar represents the variations among five independent replicates. All the difference is statistically significant by Student’s *t*-test: ^*^, *P <* 0.05; ns, not significant.

Due to the chloroplast localization of AcCaS, we investigated whether HopAU1 interacts with AcCaS on the chloroplast. We extracted chloroplasts from *N. benthamiana* leaves displaying HopAU1-GFP and AcCaS-mCherry, exhibiting GFP and AcCaS-mCherry as a control. GFP proteins were detected within chloroplasts expressing HopAU1-GFP (Fig. 4c). The data showed that HopAU1 can interact with AcCaS on the chloroplast. We first performed a protein truncation assay with Y2H to localize the protein region of AcCaS involved in HopAU1 binding. Five truncation variants of AcCaS (AcCaS^△cTP^, AcCaS-N1, AcCaS-N2, AcCaS-N3, and AcCaS-RHOD; Fig. S5) were generated and incorporated into the pGBKT7 yeast expression vector. These elements were introduced into the Y2HGold strain containing pGADT7-HopAU1. Y2H assays revealed that the amino acid region 99 to 142 of AcCaS plays a crucial role in its association with HopAU1 (Fig. 4d).

To further clarify the interaction site, we have utilized AlphaFold3 modeling to predict the structure of the AcCaS-HopAU1 complex. The modeling predicted that, within the interaction region spanning amino acids 99–142, Asn-121 of AcCaS was predicted to interact with HopAU1 (Fig. S6). Based on this, we generated a mutant version of AcCaS, termed AcCaS^N121A^, where Asn-121 was replaced with alanine (Ala), and further examined the interaction between AcCaS^N121A^ and HopAU1. The results of protein structure comparison showed that the Asn-121 to Ala substitution does not cause significant structural alterations to AcCaS (Fig. S7). Thus, we conducted LCI to further sufficiently verify their interaction. The results showed no detectable fluorescence signal in leaves expressing AcCaS^N121A^-nLuc and HopAU1-cLuc, but a clear fluorescence signal was detected in leaves expressing AcCaS-nLuc and HopAU1-cLuc (Fig. 4e). In summary, HopAU1 interacts with AcCaS on the chloroplast, the Asn-121 residue of AcCaS playing a key role in this interaction.

**Figure 6:**
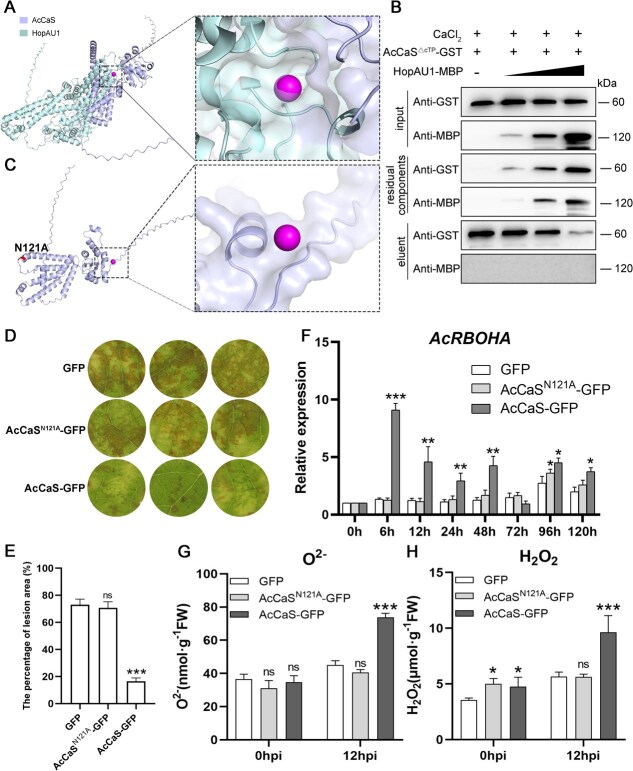
HopAU1 induces kiwifruit susceptibility to *Psa* by competing with Ca^2+^ for binding to the Asn-121 residue of AcCaS. (A) 3D structure of HopAU1, AcCaS and Ca^2+^ by AlphaFold3 modeling. (B) HopAU1 competes with Ca^2+^ to bind AcCaS. HopAU1-MBP (0, 20, 100, 500 μg) and AcCaS^△cTP^-GST were immobilized onto the phenyl-Sepharose column in the presence of 0.5 mM Ca^2+^, AcCaS^△cTP^-GST as a control. Input, the protein was loaded onto the column before beginning; residual components, flowing through the column upon loading; eluent, eluted from the column using EGTA. (C) 3D structure of AcCaS^N121A^ and Ca^2+^ by AlphaFold3 modeling. (D) Phenotypic observation of the kiwifruit leaves transiently overexpressing GFP, AcCaS^N121A^-GFP and AcCaS-GFP at 5 dpi. (E) The lesion area percentage of the kiwifruit leaves transiently overexpressing GFP, AcCaS^N121A^-GFP and AcCaS-GFP at 5 dpi. (F) Relative expression levels of ROS related genes of the kiwifruit leaves expressing GFP, AcCaS^N121A^-GFP and AcCaS-GFP at different times. (G, H) The content of O^2−^ and H_2_O_2_ in the kiwifruit leaves transiently overexpressing GFP, AcCaS^N121A^-GFP and AcCaS-GFP at 12 hpi. The error bar represents the variations among five independent replicates. All the difference is statistically significant by Student’s *t*-test: ^*^, *P <* 0.05; ^**^, *P <* 0.01; ^***^, *P <* 0.001; ns, not significant.

**Figure 7:**
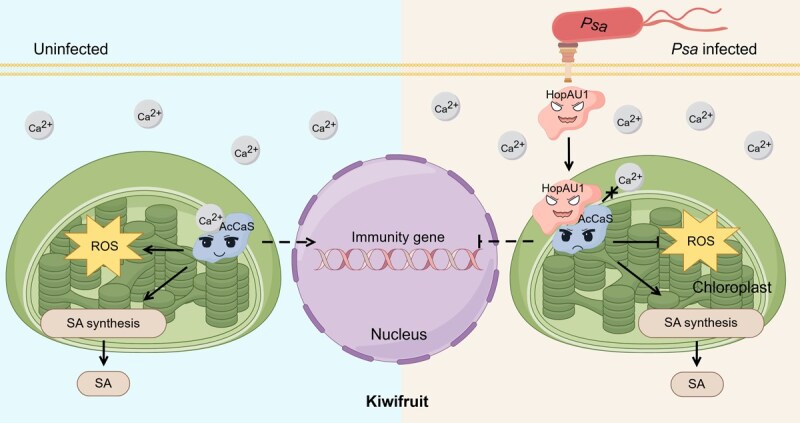
The functioning model of the HopAU1-Ca^2+^-AcCaS pathway during *Psa*–kiwifruit interaction.

### HopAU1 contributes to *Psa* virulence in an AcCaS-dependent manner

HopAU1 is a plasmid-borne effector widely distributed and evolutionarily conserved among *P. syringae* [27]. To determine the extent that HopAU1 contributes to the virulence of *Psa*, we generated HopAU1 deletion mutants (Δ*hopAU1*) in *Psa*. A *hopAU1* mutant was inoculated onto susceptible kiwifruit cultivar *A. chinensis* var. *chinensis* ‘Hongyang’. *Psa*Δ*hopAU1* produced reduced disease symptoms compared with *Psa* at 5 dpi (Fig. 5a). *Psa*Δ*hopAU1* was significantly reduced in its ability to grow in kiwifruit leaves, compared with *Psa* (Fig. 5b). Thus, HopAU1 contributes to the virulence of *Psa*. To further investigate the relationship between the virulence of HopAU1 and AcCaS, we transiently silenced AcCaS using the pFGC5941 vector and subsequently inoculated with *Psa* and *Psa*Δ*hopAU1*. The RNAi system performed effectively, as evidenced by the significant reduced expression of *AcCaS* at 4 dpi (Fig. S8). There were no significant differences in disease symptoms (Fig. 5c) or bacterial biomass (Fig. 5d) at 5 dpi. These results indicate that the virulence function of HopAU1 relies on AcCaS.

### HopAU1 competes with Ca^2+^ to bind AcCaS

AcCaS exhibits Ca^2+^-binding activity (Fig. 1d and e). Therefore, we investigated whether the interaction between HopAU1 and AcCaS affects Ca^2+^-binding ability of AcCaS. The predictions using AlphaFold3 model revealed that AcCaS fails to bind Ca^2+^ in the presence of HopAU1 (Fig. 6a). To investigate the impact of HopAU1 on the Ca^2+^-binding ability of AcCaS, we measured Ca^2+^-binding affinity of AcCaS^△cTP^-GST in the presence of HopAU1-MBP using MST assay. The results showed that AcCaS^△cTP^-GST could no longer bind Ca^2+^ when HopAU1 is present (Fig. S9).

Phenyl-sepharose-binding assay showed that AcCaS^△cTP^-GST bound to the hydrophobic matrix phenyl-sepharose in the presence of Ca^2+^. Nevertheless, upon the inclusion of HopAU1-MBP into the buffer, AcCaS^△cTP^-GST no longer interacted with the phenyl-sepharose column. With MBP added to the buffer as a control, AcCaS^△cTP^-GST still interacted with the phenyl-sepharose column. This indicates that HopAU1-MBP inhibits the interaction between AcCaS^△cTP^-GST and phenyl-sepharose in the presence of Ca^2+^ (Fig. S10). Therefore, we investigated whether it competes with Ca^2+^ to bind AcCaS. The result shows that less AcCaS is pulled down in the phenyl-sepharose-binding assay following the addition of increasing amounts of HopAU1 (Fig. 6b), indicating that HopAU1 can interfere with the interaction between AcCaS and Ca^2+^. Overall, these findings indicate that HopAU1 inhibits the Ca^2+^-binding ability of AcCaS through competitive interaction.

### The disease resistance function of AcCaS depends on the Ca^2+^-binding site Asn-121

HopAU1 competes with Ca^2+^ to bind AcCaS (Fig. 6b) and specifically interacts with the Asn-121 residues of AcCaS (Fig. 4e). This competitive binding mechanism suggests a potential interference with AcCaS calcium-binding capabilities. Therefore, utilizing AlphaFold3 modeling, we demonstrated that AcCaS^N121A^ is unable to bind Ca^2+^ (Figs 6c and S11). We created a mutant version of AcCaS^△cTP^, termed AcCaS^△cTP-N121A^, where the Asn were substituted by alanine (Ala). In MST assay, AcCaS^△cTP-N121A^ no longer binds to Ca^2+^ (Fig. S12A), compared with the control AcCaS^△cTP^ (Fig. S12B). This indicates that the Ca^2+^-binding ability of AcCaS depends on Asn-121.

Furthermore, to investigate whether the Ca^2+^-binding ability of AcCaS influences its role in disease resistance, we performed a functional analysis of AcCaS^N121A^. An overexpression pCAMBIA1302 vector was used to express AcCaS and AcCaS^N121A^. GFP represents the kiwifruit with the empty vector. AcCaS-GFP was used as the positive control and GFP as the negative control. The overexpression system performed effectively, as evidenced by the significant increase expression of *AcCaS* and *AcCaS^N121A^* at 2 dpi (Fig. S13). After inoculation with *Psa*, the infected leaves of AcCaS^N121A^-GFP exhibited milder disease progression and the lesion area percentage was not significant with GFP but a significantly higher lesion area percentage compared to AcCaS at 5 dpi (Fig. 6d and e). Similarly, we quantified ROS levels after overexpression AcCaS^N121A^-GFP. After inoculated with *Psa* for 12 h, overexpressing AcCaS^N121A^-GFP not significant ROS contents compared to WT but lower ROS contents compared to AcCaS (Fig. 6g and h). The expression of ROS related gene *AcRBOHA* was not significant at 6, 12, 24, 48, 72, and 120 hpi (Fig. 6f). This indicates that the ability of AcCaS to bind Ca^2+^ is crucial for its disease-resistance function.

## Discussion

Through prolonged co-evolutionary processes, plants and microbial pathogens have established intricate infection and defense mechanisms, consistent with the Zig-Zag model of plant–pathogen interactions [28]. The plant immune system comprises two primary defense tiers: PTI, initiated through membrane-localized pattern recognition receptors detecting conserved pathogen-associated molecular patterns, and effector-triggered immunity (ETI), activated by intracellular resistance (R) proteins that recognize pathogen-secreted effector molecules [29]. Both PTI and ETI trigger downstream signaling cascades, including ROS bursts, Ca^2+^ fluxes, activation of mitogen-activated protein kinase cascades, transcriptional upregulation of pathogenesis-related genes, and localized deposition of callose-rich cell wall reinforcements [30].

Among these, Ca^2+^ serves as a critical second messenger in plants, playing a key role in responding to environmental changes and resisting pathogen infections. A rapid and transient increase in cytosolic Ca^2+^ concentration is one of the earliest cellular responses during plant immunity. Changes in intracellular Ca^2+^ levels generate Ca^2+^ signals [31], which are recognized by specific calcium sensors or receptors, leading to subsequent transcriptional and metabolic responses. Most calcium sensors recognize these signals through EF-hand motifs [32]. Calcium-sensing receptor (CaS) is a plant-specific regulatory protein localized to the stromal side of chloroplast thylakoid membranes, operates as a low-affinity/high-capacity Ca^2+^ buffer through a unique Ca^2+^-binding mechanism independent of canonical EF-hand motifs. This membrane-associated sensor decodes extracellular calcium fluctuations into intracellular signaling events by modulating stromal Ca^2+^ homeostasis, thereby coordinating downstream phosphorylation cascades that mediate plant adaptation to environmental challenges. CaS is essential for both PTI and ETI [22]. In *Arabidopsis*, SsITL from *Sclerotinia sclerotiorum* suppresses SA accumulation by interacting with CaS, thereby promoting infection [23]. In our study, AcCaS positively regulates kiwifruit immune responses, its ability to bind Ca^2+^ and confer disease resistance depends on the Asn-121 residue. Unlike in *Pst* DC3000, HopAU1 from *Psa* targets the Asn-121 residue of AcCaS, competitively binding with Ca^2+^ to inhibit its disease resistance function and thereby suppressing kiwifruit immune responses.

Recent studies have shed light on the interactions between *Psa* and kiwifruit. For instance, HopZ5 is recognized by NbPTR1, and overexpression of NbPTR1 has been indicated to significantly enhance kiwifruit resistance [33]. Furthermore, HopZ5 interacts with AcGF14C, which plays a key role in boosting kiwifruit immunity [34]. Previous research has shown that HopAU1 targets the calcium-sensing receptor (CaS) in *N. benthamiana* leaves to regulate tobacco immunity [26]. Effectors impact host immune responses through diverse mechanisms, exhibiting either virulence or avirulence functions. However, the mechanisms by which *Psa* effectors influence chloroplast function in kiwifruit remain largely unknown. Kiwifruit phloem and leaves contain abundant chloroplasts, and during *Psa* infection, both tissues accumulate higher levels of bacteria. This suggests that chloroplasts play a critical role in plant defense responses. Understanding the interaction between HopAU1 and AcCaS in chloroplasts provides a foundation for further exploring chloroplast-mediated immunity in kiwifruit. AcCaS contains a 70-amino-acid N-terminal chloroplast transit peptide, and we found that chloroplast localization of AcCaS is essential for its immune function. Specifically, the first 22 amino acids of the N-terminus are required for its chloroplast targeting. Unlike other effectors, HopAU1 directly interacts with AcCaS in the chloroplast, disrupting AcCaS-mediated chloroplast immunity. This provides new insights into the kiwifruit–*Psa* interaction mechanism.

Among the 31 effectors identified in *Psa* [27], the functional mechanisms and target genes of most effectors remain poorly understood. Our study elucidates the mechanism by which HopAU1 modulates kiwifruit immunity and offers new mechanistic insights into the role of AcCaS in disease resistance. While AcCaS-overexpressing shows potential for enhancing disease resistance, AcCaS is widely present in both susceptible and resistant cultivars and plays a crucial role in calcium signaling. Therefore, further investigations are required to clarify its precise functions and evaluate its potential applications. In this study, we identified AcCaS is highly conserved and positively correlated with resistance to *Psa*. Our research on the interaction between AcCaS and HopAU1 has identified critical disease-resistant sites Asn-121 in AcCaS and elucidated HopAU1 competes with Ca^2+^ to bind AcCaS (Fig. 7). These findings establish a theoretical foundation for rationally designing disease-resistant molecules targeting chloroplast signaling hubs. In addition, given the limited number of disease-resistant kiwifruit cultivars, we have developed AcCaS-overexpressing plants that exhibit significantly enhanced resistance, making them valuable for further application and development. Overall, our research elucidates the mechanism of interaction between AcCaS and HopAU1 in the chloroplast of kiwifruit, contributing to a deeper understanding of plant–pathogen interactions and laying the foundation for innovative strategies to enhance host resistance to pathogens.

## Conclusion

In summary, our study characterizes *AcCaS* as a chloroplast-localized gene that positively regulates plant immunity through dual mechanisms: facilitating chloroplastic ROS burst and Ca^2+^ interaction. Importantly, the chloroplast localization of AcCaS was found essential for its immune-activating capacity. We identified that the *Psa* effector HopAU1 promotes pathogenesis through competitive binding at the Asn-121 residue of AcCaS, effectively disrupting its Ca^2+^-binding functionality within chloroplasts (Fig. 7). We confirmed the Asn-121 site as a critical determinant for both Ca^2+^ coordination and disease resistance activity. These findings elucidate a novel pathogenic strategy of immune suppression through chloroplast-targeted effector competition, while advancing our understanding of calcium-mediated defense mechanisms in kiwifruit–*Psa* interactions.

## Materials and methods

### Plant and microbe materials

The study focused on the leaves of three-year-old potted kiwifruit (*A. chinensis* var. *chinensis* ‘Hongyang’) cultivated in the greenhouse at Northwest A&F University. The strain of *Psa* was supplied by the Fruit Tree Pathogenic Mechanism and Integrated Control Group of Northwest A&F University. The strains employed in this research were propagated on LB agar plates supplemented with appropriate antibiotics at 28°C for 2 d. Subsequently, the LB liquid medium was prepared and incubated at 28°C under shaking conditions at a speed of 220 r/min for 16–20 h. The bacterial cells were obtained at a speed of 8000 rpm, diluted with sterile distilled water, the resulting solution attains a final concentration of around 1 × 10^8^ CFU/ml (OD600 = 0.1), facilitating subsequent infection experiments.

### RNA-seq analysis

RNA-seq analysis was carried out using the BMKCloud platform. The sequences were aligned with the kiwifruit reference genome (*A. chinensis* Hong Yang v3). Differentially expressed genes (DEGs) were examined utilizing the DESeq2, significant variations in expression are identified when the *P*-value is less than 0.05 and the log_2_ value (fold change) exceeds 1.

### Pathogenicity tests

The pathogenicity test used the indoor bioassay method of vacuum infiltration on kiwifruit leaf discs following the previously developed method [27]. The kiwifruit leaves used in the experiment were all annual, and all leaves between treatments and replicates were taken from the same greenhouse and maintained in a healthy state. In the case of vacuum infiltration inoculation on intact kiwifruit foliage, a surface sterilization process utilizing 0.6% sodium hypochlorite was carried out for a duration of 10 min, this is followed by three rinses using sterile water. Subsequently, the kiwifruit foliage was transformed into circular discs with a radius of 0.5 cm. A bacterial suspension with a concentration of 1 × 10^5^ CFU/ml was introduced onto leaf discs through vacuum infiltration inoculation. Each treatment was conducted with at least 15 replicates. The treated specimens were subjected to an artificial climate-controlled environment within the incubator, maintaining 16°C, with relative humidity set at 75%. The occurrence of leaves was measured at 5 dpi. The bacterial growth assay technique has been previously detailed in a previous study [35]. The incidence of the disease in kiwifruit leaf discs was determined utilizing Image J software (v1.53 t).

### Construction of insertion inactivation mutants

Genome-wide gene deletion mutants were constructed using the SacB-unmarked homologous recombination approach. Deletion strains were generated through an inactivation process utilizing a homologous, one-step vector integration strategy, employing the suicide vector pK18mobSacB.

### RNA isolation, cDNA generation, and qRT-PCR analysis

The leaves of kiwifruit were collected and processed into a powder using liquid nitrogen, which facilitated the extraction of RNA. Total RNA was isolated from plant samples utilizing the Quick RNA Extraction Kit (Huayueyang, Beijing, China). The initial cDNA strand was generated utilizing the Revert Aid First Strand cDNA Synthesis Kit (Thermo Fisher Scientific, Waltham, Massachusetts, United States). qRT-PCR was conducted utilizing the Light Cycler 96 System (Roche, Mannheim, Germany), employing the Cham QSYBR Color qPCR Master Mix (Vazyme, Nanjing, China). The comparative gene expression was investigated utilizing the 2^−ΔΔCT^ approach [36]. There was a total of three technical replicates for each treatment and each experiment was conducted a total of three times. Primers used for the qRT-PCR analysis are shown in Table S3.

### Transient expression experiment in kiwifruit

The pCAMBIA1302 vector was employed for transient overexpression, while the pFGC5941 vector was utilized for transient silencing. Primers used to construct the expression vectors are listed in Table S2. *Agrobacterial* cells harboring the pCAMBIA1302 and pFGC5941 constructs were reconstituted in a buffer solution comprising 10 mM MgCl_2_, 10 μM AS, and 10 mM 2-(N-morpholino) ethanesulfonic acid (MES), a solution with an OD600 value of 0.5 was introduced into the leaf discs (1 cm in diameter) via vacuum infiltration at a pressure of 0.1 MPa for a duration of 10 s. Leaf discs were grown on 0.5% agar plates at 28°C. Gene expression levels were investigated through qRT-PCR, with samples being obtained at 2 d for overexpression and 4 d for silencing.

### Isolation and observation of chloroplasts

Under the influence of *Agrobacterial*, three *N. benthamiana* leaves were collected after 48 hpi and subsequently divided into smaller fragments using a mortar. The separation of chloroplasts was achieved using the Chloroplast extraction kit (Solarbio, Beijing, China). Subsequently, 200 μl of the supernatant along with the sediment was mixed with 200 μl of suspension buffer (0.35 M NaCl) and examined using an Olympus BX-51 fluorescence microscope (ocular: 10×; objective: 20×).

### Bioinformatic analysis

The examination of the domain structure of AcCaS was conducted utilizing the SMART tool (http://smart.embl.de/). The chloroplast transit peptide of AcCaS was determined through the TargetP1.1 analysis (http://www.cbs.dtu.dk/services/TargetP/). The analysis of transmembrane domains in AcCaS was conducted utilizing the TMHMMServerv.2.0 (http://www.cbs.dtu.dk/services/TMHMM/). AcCaS homologs were obtained from the NCBI database through BLASTp search. The protein sequences were aligned using ClustalW and subsequently visualized utilizing ESPript 3.0 (ESPript 3.x/ENDscript 2.x). MEGA11, a powerful bioinformatics software, was employed for the construction of a phylogenetic tree utilizing the neighbor-joining method. This process involved conducting a comprehensive 1000-replicate bootstrap test to ensure the reliability and accuracy of the resulting tree.

### Subcellular localization

In the process of localization, *Agrobacterial* bearing the appropriate constructs were redispersed in a buffer solution (10 mM MgCl_2_, 10 μM AS, 10 mM MES) at OD_600_ of 0.5 and introduced into 4-week-old *N. benthamiana* leaves. At 48 hpi, GFP and chloroplast fluorescence were detected using confocal microscopy FV3000 (ocular: 10×; objective: 20×).

### Yeast two-hybrid assays, LCI and MBP pull-down

In the yeast two-hybrid (Y2H) assays, various segments of AcCaS were integrated into pGBKT7, resulting in the formation of AcCaS-BD constructs. HopAU1 was integrated into the pGADT7 vector, resulting in the creation of the HopAU1-AD fusion protein. The Y2H Gold yeast strain was utilized to conduct Y2H assays. The yeast transformation process was carried out in accordance with the detailed guidelines provided by the manufacturer. Transformants obtained on SD/-Trp/-Leu medium were cultivated on SD/-Trp/-Leu/-His/-Ade medium supplemented with X-*a*-galactosidase (X-*a*-gal) for growth.

In the LCI assays, Nluc and Cluc constructs were encapsulated within *Agrobacterial*, which were subsequently homogeneously blended and introduced into *N. benthamiana* leaves, as previously mentioned [37]. On the 2 d following infiltration, the conditioned leaves underwent an additional infiltration with 0.5 mM D-Luciferin (Bivision, Nanjing, China) the participants were kept in the dark for a duration of 5 min. The images were then visualized using multispectral dynamic fluorescence microscopy (PlantView100).

In the MBP pull-down assays, pGEX-4T-1 and pMal-c4X-MBP plasmids were introduced into *E. coli* strain BL21 (DE3). Recombinant protein expression was triggered using 0.1 mM IPTG at 16°C for 18–20 h. Bacterial cell lysates were subjected to incubation with GST-tagged Purification Resin (P2251, Beyotime, Shanghai, China) and Dextrin beads (Smart-Lifesciences, Jiangsu, China). Input and pull-down fractions were subjected to immunoblotting analysis using anti-GST (M20007, Abmart, Shanghai, China) and anti-MBP (M20051, Abmart, Shanghai, China) antibodies, respectively.

### Observation of chloroplast ROS

We employed 2′7′-dichlorodihydrofluorescein diacetate (H2DCF-DA) as a sensing tool to identify ROS generated in *N. benthamiana*. The protein was briefly present in *N. benthamiana.* After 2 dpi, a concentration of 10 μM H2DCF-DA (Sigma-Aldrich, product code D6883) was administered to the leaves via a syringe at 8 h. Subsequently, the specimens underwent visual examination and imaging using a laser-scanning confocal microscope FV3000 (ocular: 10×; objective: 20×) under illumination with a laser at 488 nm and fluorescence emission within the range of 512–527 nm.

### Detection of ROS

Kiwifruit leaf discs, subjected to vacuum infiltration with *Psa*, were cultured at 16°C. At 0 and 12 h, leaf discs with a mass of 0.1 grams were gathered for processing in liquid nitrogen, which would be used for subsequent experiments. H_2_O_2_ and superoxide anion (O^2−^) levels were measured utilizing the superoxide dismutase assay kit (Suzhou Grace Biotechnology, Jiangsu, China) and O^2−^ scavenging capacity kit (Suzhou Grace Biotechnology, Jiangsu, China).

Kiwifruit leaf discs were immersed in a DAB aqueous solution (containing 1 mg/ml DAB, pH 3.8) and subjected to light exposure for a duration of 8 h. Subsequently, the leaves of kiwifruit were subjected to a 10 min immersion in 95% ethanol, followed by decolorization, and were examined under an optical microscope (BX53, Olympus) for detailed imaging.

### MicroScale thermophoresis

The MST assays were carried out according to the methodology outlined in Nie *et al.* [38]. The refined proteins were tagged utilizing the Monolith™ RED-NHS Second Generation Protein Labeling Kit (appropriate for application with Monolith™ NT.115 series and NT. Automated series instruments featuring red light sensors, MO-L011) sourced from NanoTemper (Germany). MST assays were conducted utilizing the Monolith™ NT.115 Microthermophoresis Instrument (NanoTemper, Germany) in collaboration with capillaries from the Monolith™ NT.115 Series (MO-K022), which are also provided by NanoTemper (Germany).

### Phenyl-sepharose-binding assay

The AcCaS^△cTP^-GST and HopAU1-MBP protein (each 0.1 mg) were introduced onto a column filled with 0.5 ml of phenyl-sepharose, concurrently with the presence of 0.5 mM Ca^2+^. Following comprehensive rinsing, the extraction was performed utilizing a solution comprising 5 mM EGTA. Fractional samples (0.1 milliliters each) were gathered, and these fractions underwent analysis through 10% sodium dodecyl sulfate-polyacrylamide gel electrophoresis (SDS-PAGE).

### Generation of transgenic kiwifruit plants

The AcCaS coding sequence was successfully integrated into the pCambia1302 vector, with the 35S promoter acting as the driving force behind its expression. The resulting constructs, pCambia1302-AcCaS, were converted into *A. chinensis* var. *chinensis* ‘Hongyang’, has been utilized with the *Agrobacterium* strain EHA105.

### 3D structure

The protein structure was forecast using AlphaFold3 (https://golgi.sandbox.google.com/) and depicted utilizing PyMOL.

### Statistical analysis

Utilizing Graphpad Prism 8, the development of bar charts and statistical evaluations was achieved. *P*-values were determined using the two-tailed Student’s *t*-test method.

## Supplementary Material

Web_Material_uhaf230

## Data Availability

The raw RNA-Seq data that support the findings of this study are openly available in NCBI at (https://www.ncbinlm.nih.gov/sra/PRINA1054973), reference number (PRJNA1054973). The sequence data in this study can be found in the Hong Yang v3 genome (https://kiwifruitgenome.atcgn.com) under accession numbers in the article and Supporting Information Tables S1. All the relevant data supporting the findings of this study are available in the article and supplementary data.
