# Supplementary material for: Calcium-sensing receptor AcCaS regulates chloroplast immunity in kiwifruit by competitively binding with Ca2+ or the Psa effector
Source: Hortic Res. 2025 Sep 3;12(12):uhaf230. doi: 10.1093/hr/uhaf230 (PMC12682068; doi:10.1093/hr/uhaf230)
Supplement: Web_Material_uhaf230 [file web_material_uhaf230.zip › Supplementary Information-clean.docx]

**Supplementary Information**

**Title**

Calcium-sensing receptor AcCaS regulate chloroplast immunity in kiwifruit by competely binding with Ca^2+^ or *Psa* effector

**Authors**

Rui Li^†^, Yali Zhang^†^, Xiaofei Du, Xinxin Wang, Wei Liu*, Lili Huang*

^†^ These authors equally contributed to this work.

**Affiliations**

State Key Laboratory of Crop Stress Resistance and High-Efficiency Production,

College of Plant Protection, Northwest A&F University, Yangling, Shaanxi 712100, China.

*** Correspondence authors**

Lili Huang, [huanglili@nwsuaf.edu.cn](mailto:huanglili@nwsuaf.edu.cn%20(L.H.)),

Wei Liu, wliu@nwsuaf.edu.cn.


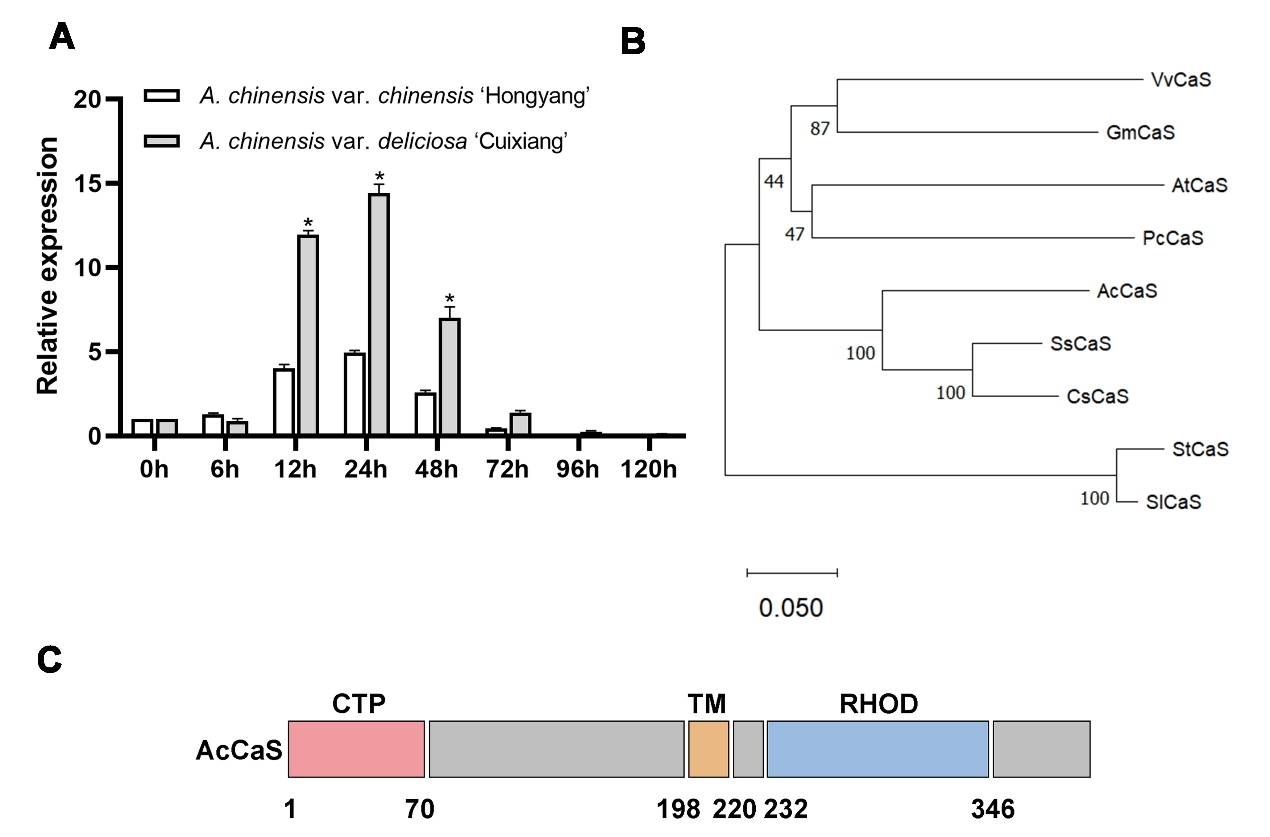


**Figure S1. The function and phylogenetic analysis of AcCaS.** (A) Expression analysis of *AcCaS* across ‘Hongyang’ and ‘Cuixiang’ at 0, 6, 12, 24, 48, 72, 96, 120hpi. (B) Phylogenetic analysis of AcCaS. (C) Structure diagram of AcCaS.


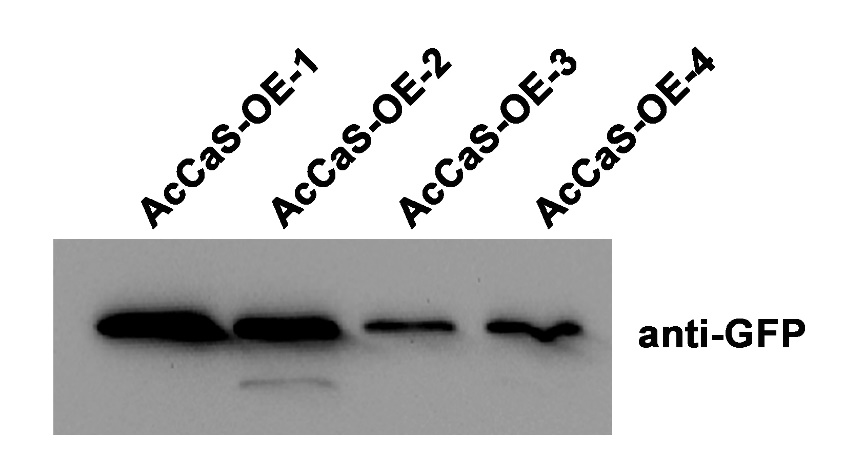


**Figure S2. Overexpression of AcCaS in transgenic kiwifruit.**


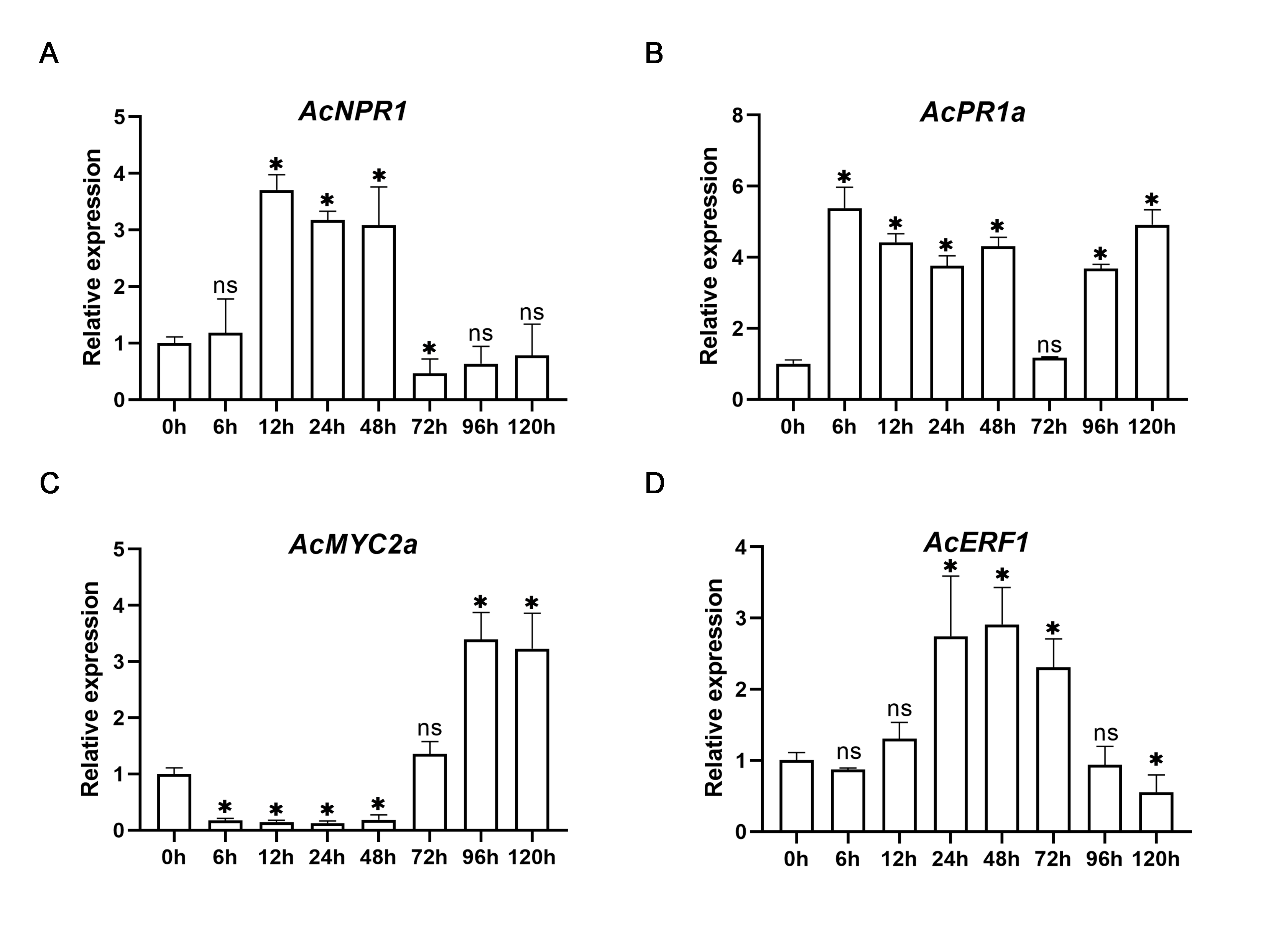


**Figure S3. Analyze the expression of different pathway genes in AcCaS.** (A-B) The relative expression levels of the salicylic acid (SA) signalling pathway-related genes. (C) The relative expression levels of the jasmonic acid (JA) signalling pathway-related genes. (D) The relative expression levels of the ethylene (ET) signalling pathway-related genes. The error bar represents the variations among three independent replicates. All the difference is statistically significant by Student's *t*-test: *, *P* < 0.05; ns, not significant.


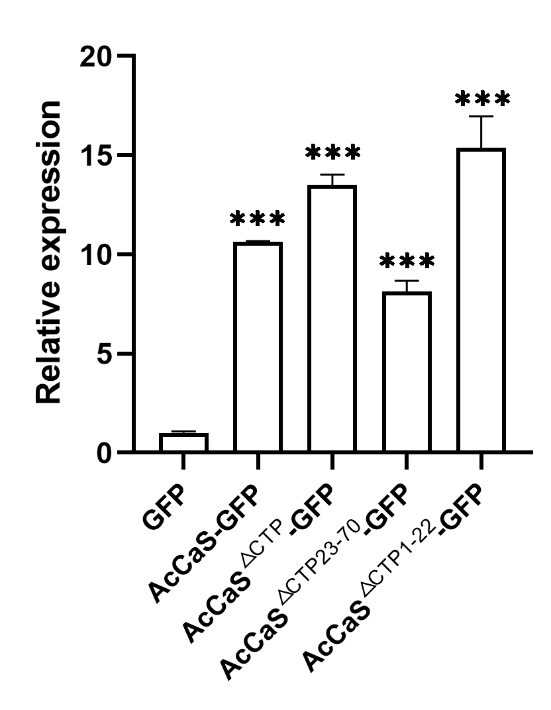


**Figure S4. The expression of *AcCaS* in overexpression kiwifruit at 2 dpi.** The error bar represents the variations among three independent replicates. All the difference is statistically significant by Student's *t*-test: ***, *P*<0.001.


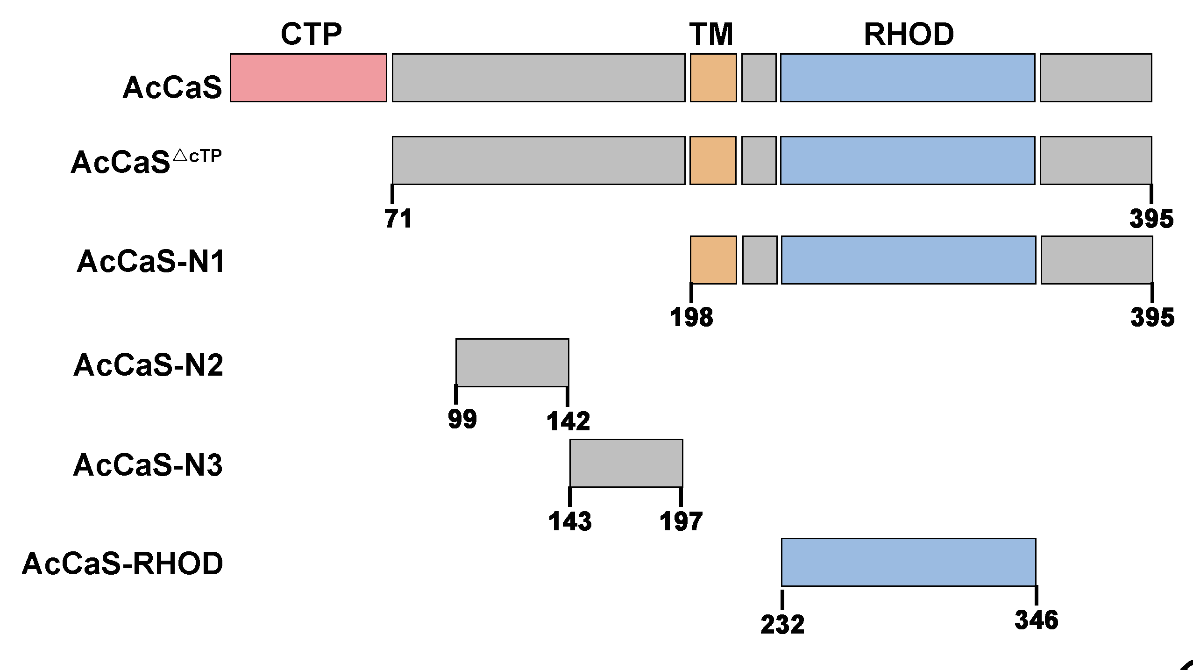


**Figure S5. Schematic diagram of AcCaS truncation mutants.** Numbers represent the amino acid location.


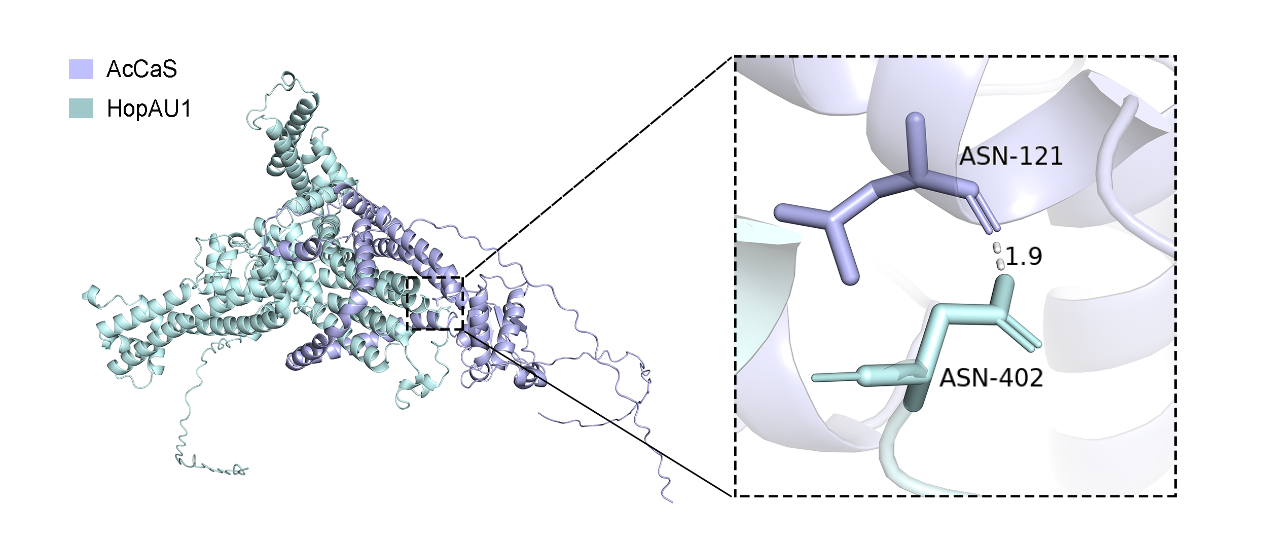


**Figure S6. 3D structure of HopAU1 and AcCaS by AlphaFold3 modeling.**


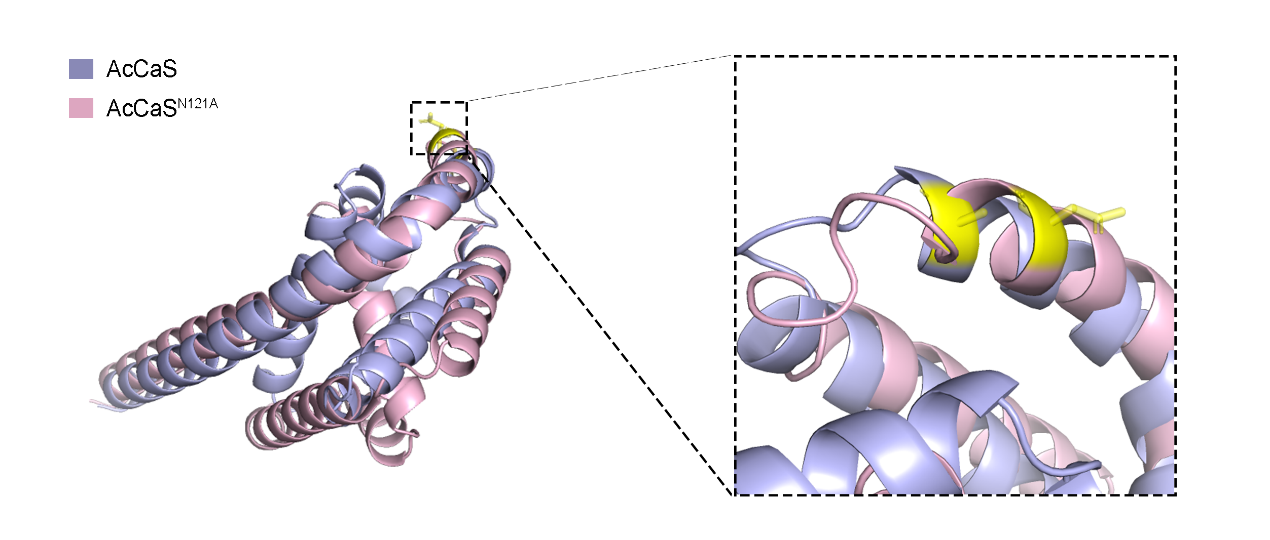


**Figure S7. 3D structure of AcCaS and AcCaS^N121A^ by AlphaFold3 modeling.**


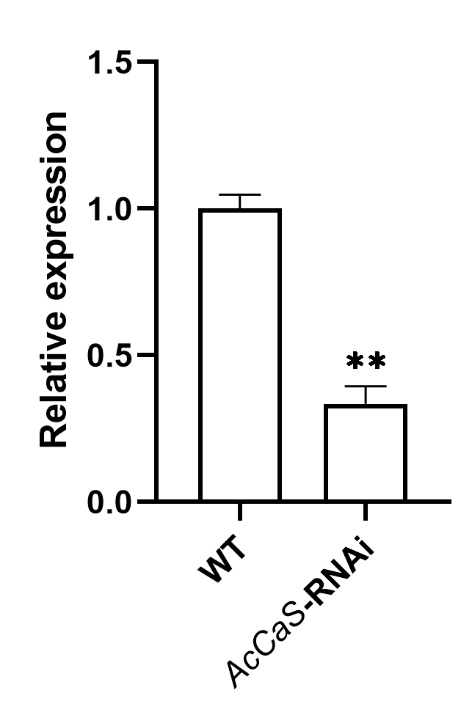


**Figure S8. The expression of *AcCaS* in RNAi kiwifruit at 4 dpi.** The error bar represents the variations among three independent replicates. All the difference is statistically significant by Student's *t*-test: **, *P*<0.01.


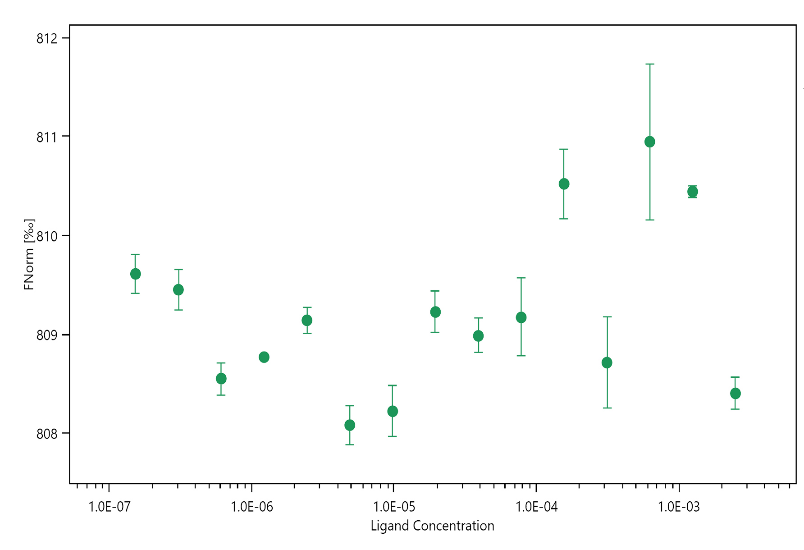


**Figure S9. HopAU1 inhibits the binding of AcCaS to Ca^2+^.** Using MST assays, the AcCaS^△cTP^-GST, HopAU1-MBP and Ca^2+^ were contained in NT standard capillaries. The error bar represents the variations among three independent replicates.


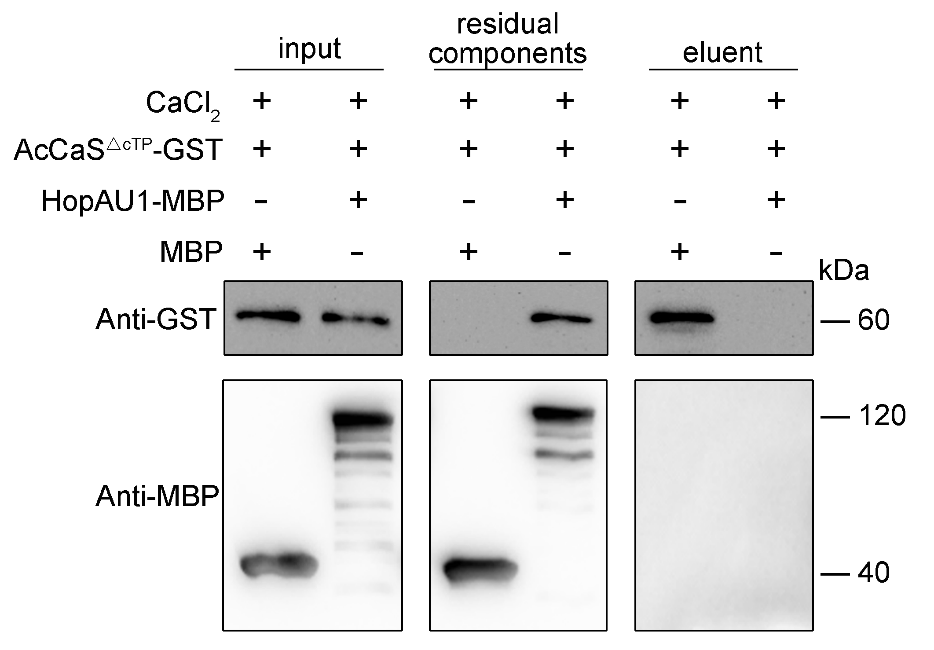


**Figure S10. HopAU1 inhibited the binding of AcCaS to a phenyl-Sepharose column in the presence of Ca^2+^.** HopAU1-MBP and AcCaS^△cTP^-GST were loaded onto the phenyl-Sepharose column in the presence of 0.5 mM Ca^2+^, MBP and AcCaS^△cTP^-GST as a control. Then eluted using a buffer containing 5mMEGTA. All fractions were analyzed by immunoblotting.


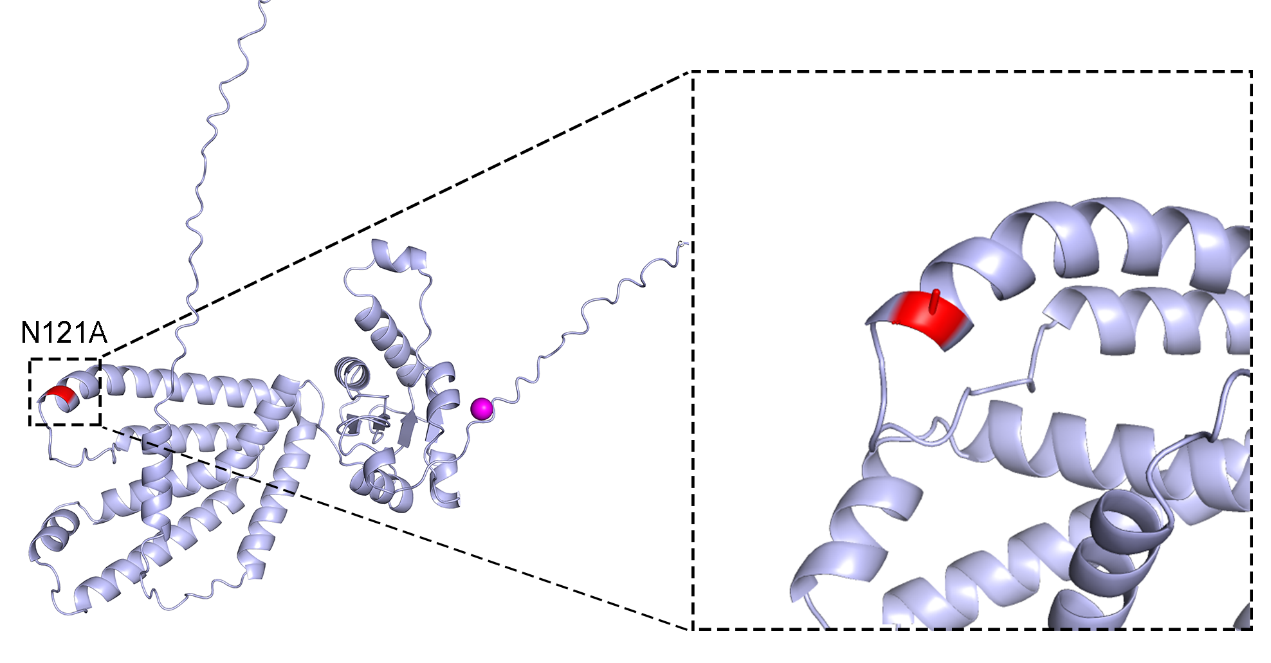


**Figure S11. 3D structure of Ca^2+^ and AcCaS^N121A^ by AlphaFold3 modeling.**


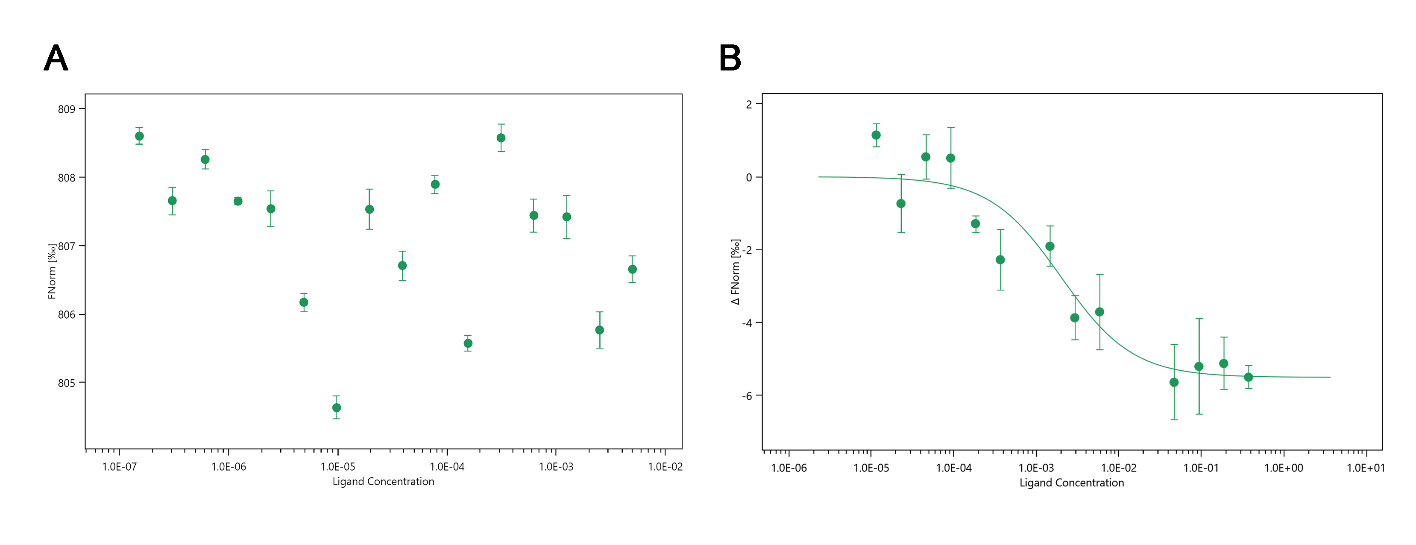


**Figure S12. MST assays show that AcCaS^△cTP-N121A^ and AcCaS^△cTP^ interacts with Ca^2+^.** (A) MST assays show that AcCaS^△cTP-N121A^ does not interacts with Ca^2+^. (B) MST assays show that AcCaS^△cTP^ interacts with Ca^2+^. The Ca²⁺ and AcCaS^△cTP-N121A^-GST were contained in NT standard capillaries.


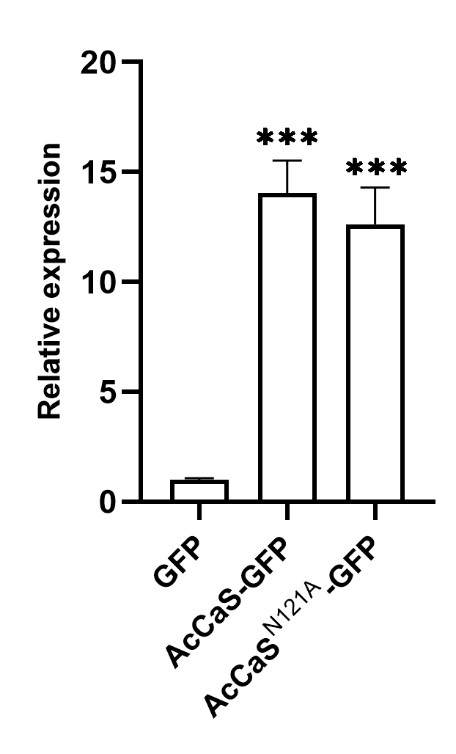


**Figure S13. The expression of *AcCaS* and *AcCaS^N121A^* in overexpression kiwifruit at 2 dpi.** The error bar represents the variations among three independent replicates. All the difference is statistically significant by Student's *t*-test: ***, *P*<0.001.
